# Editorial: Superlubricity across the scales

**DOI:** 10.3389/fchem.2022.1063330

**Published:** 2022-10-26

**Authors:** Mehmet Z. Baykara, Diana Berman, Andreas Rosenkranz

**Affiliations:** ^1^ Department of Mechanical Engineering, University of California Merced, Merced, CA, United States; ^2^ Department of Materials Science and Engineering, University of North Texas, Denton, TX, United States; ^3^ Department of Chemical Engineering, Biotechnology and Materials, FCFM, Universidad de Chile, Santiago, Chile

**Keywords:** friction, nanomechanics, nanotribology, superlubricity, tribology

Those who were into computer games in the late 1990s may remember the famous space strategy game *Sid Meier’s Alpha Centauri*, where one of the critical technologies that could be attained by a given civilization was called *frictionless surfaces*. While the idea of frictionless surfaces and the associated implications of vanishing energy losses during mechanical motion have been part of science fiction culture, scientists in the real world work toward realizing this ambitious goal that was once thought to be unattainable.

A key term associated with this line of research is “superlubricity”, which implies ultra-low friction forces and an effective coefficient of friction below 0.01 (Baykara et al., 2018; Ayyagari et al., 2022). A myriad of approaches can be taken toward realizing such ultra-low levels of friction, ranging from magnetic levitation and the use of liquid lubricants under elastohydrodynamic lubrication all the way to seemingly exotic ideas such as “structural superlubricity” of solid material interfaces. The latter is especially interesting from a fundamental point of view as it implies that nearly frictionless conditions should emerge whenever relative motion occurs at an interface that is atomically flat, molecularly clean, and consisting of two surfaces with atomic structures in an incommensurate contact. Despite the theoretically straightforward geometric arguments that give rise to the idea of structural superlubricity, its experimental realization is still limited to a small number of examples (Hod et al., 2018). There is still a lot to learn with respect to its physical limits, particularly in terms of environmental (e.g. temperature, humidity) and operating (e.g. sliding speed, contact size) conditions.

The overarching goal of the research topic titled “Superlubricity Across the Scales” is to provide a snapshot of the latest developments in this rapidly accelerating field of research. This is achieved by four articles that describe progress in diverse areas of superlubricity. Gao and Müser direct their attention to a material system, gold islands on graphite, that attracted recent interest due to its structurally superlubric nature under ambient conditions (Cihan et al., 2016). By way of molecular dynamics (MD) simulations, they explore the mechanisms of kinetic friction at the interface of two materials and the role of the boundary conditions used in the simulations. The second article on the research topic, written by Wang et al., also focuses on the concept of structural superlubricity, this time from the perspective of loading area dependence. By performing MD simulations, the researchers reveal two distinct dependencies of friction on normal load, with the determining factor being the size of the loading area. Moving away from the concept of structural superlubricity and the hard, physical material systems that are often part of related research, Chau et al. present results on the superlubricity of pH-responsive hydrogels, whereby they discover tunable lubricative properties as a function of pH levels. This work highlights the potential importance of superlubricity for materials used in biomedical applications. Finally, the contribution by Sui et al. investigates the lubricative influence of metal-organic framework (MOF) nanoparticles as additives in water-lubricated contacts between ceramic pairs.

Based on its current trajectory, it is expected that fundamental superlubricity research will continue to flourish in the near future, and proof-of-principle applications that rely on superlubricious mechanical contacts with minimal energy dissipation will emerge soon. The eventual extension of the concept to more conventional engineering systems, which would require significant technological innovations based on fundamental discovery, would then have significant implications for energy savings and sustainability of mechanical systems.
